# Efficacy of triple antiemetic therapy (palonosetron, dexamethasone, aprepitant) for chemotherapy-induced nausea and vomiting in patients receiving carboplatin-based, moderately emetogenic chemotherapy

**DOI:** 10.1186/s40064-016-3769-x

**Published:** 2016-12-07

**Authors:** Toshimichi Miya, Kunihiko Kobayashi, Mitsunori Hino, Masahiro Ando, Susumu Takeuchi, Masahiro Seike, Kaoru Kubota, Akihiko Gemma, East Japan Chesters Group

**Affiliations:** 1 Department of Pulmonary Medicine/Medical Oncology, Nippon Medical School, Tamanagayama Hospital, 1-7-1 Nagayama, Tama, Tokyo 206-8512 Japan; 2 Department of Pulmonary Medicine, Saitama Medical University International Medical Center, Hidaka, Saitama Japan; 3 Department of Pulmonary Medicine, Nippon Medical School, Chiba Hokusoh Hospital, Inzai, Chiba Japan; 4 Department of Pulmonary Medicine, Jizankai Medical Foundation Tsuboi Cancer Center Hospital, Kohriyama, Fukushima Japan; 5 Department of Pulmonary Medicine and Oncology, Graduate School of Medicine, Nippon Medical School, Tokyo, Japan

**Keywords:** Triple antiemetic therapy, Moderately emetogenic chemotherapy, Palonosetron, Aprepitant, Dexamethasone

## Abstract

**Background:**

Chemotherapy-induced nausea and vomiting (CINV) is a major adverse toxicity of cancer chemotherapy. Recommended treatments for prevention of CINV vary among published guidelines, and optimal care for CINV caused by moderately emetogenic chemotherapy has not been established. This study assessed the efficacy and safety of triple antiemetic therapy comprising palonosetron, dexamethasone and aprepitant for carboplatin-based chemotherapy. Chemotherapy-naïve patients with lung cancer scheduled for a first course of a carboplatin-containing regimen formed the study cohort. Patients were pretreated with antiemetic therapy comprising palonosetron (0.75 mg, i.v.) and dexamethasone (9.9 mg, i.v.) on day 1, and aprepitant (125 mg, p.o.) on day 1 followed by 80 mg on days 2 and 3. Primary endpoint was the proportion of patients who did not experience vomiting and did not require rescue medication [complete response (CR)] in the acute phase (0–24 h), late phase (24–168 h) and overall. Secondary endpoint was the proportion of patients who experienced no vomiting episodes and no more than mild nausea without the need for rescue medication [complete control (CC)].

**Results:**

Prevalence of a CR during the acute phase, delayed phase, and overall was 100, 91.9 and 91.9%, whereas that of CC was 100, 84.4 and 84.4%, respectively. The most common adverse event was mild constipation; severe adverse events related to antiemetic treatment were not observed.

**Conclusion:**

Triple antiemetic therapy comprising palonosetron, dexamethasone and aprepitant shows excellent effects in the prevention of CINV in patients receiving a carboplatin-containing regimen.

## Background

Despite the introduction of antiemetic treatments such as corticosteroids, 5-hydroxytryptamine-3 (5-HT3) receptor antagonists, and neurokinin-1 (NK-1) receptor inhibitors, chemotherapy-induced nausea and vomiting (CINV) remains a major toxicity of cancer chemotherapy that reduces the quality of life (QOL) of cancer patients.

In guidelines relating to clinical practice, chemotherapy drugs are classified according to their “emetogenicity”. Guidelines state that antiemetic treatments should be adopted according to the category of emetic risks, but there are some differences in the recommendations for moderately emetogenic chemotherapy (MEC) among such guidelines. Guidelines set by the American Society of Clinical Oncology recommend a two-drug combination comprising palonosetron and dexamethasone as antiemetic treatment for MEC (Basch et al. 2011). In contrast, guidelines set by the National Comprehensive Cancer Network recommend a double combination of a 5-HT3 receptor antagonist and dexamethasone, and an additional NK-1 inhibitor (e.g., aprepitant) is recommended for patients treated with a carboplatin- or irinotecan-containing regimen (Ettinger et al. 2012). The standard of care indicated by these guidelines has improved the prevalence of emetic events markedly, but almost half of patients continue to suffer acute and delayed CINV after MEC (Di Maio et al. 2015). Antiemetic therapy should aim to minimize or eliminate CINV in an optimal manner in all cancer patients, so the methods of CINV control can be improved further.

Recently, the Multinational Association of Supportive Care in Cancer (MASCC) and the European Society of Medical Oncology (ESMO) updated the guidelines for prevention of chemotherapy- and radiotherapy-induced nausea and vomiting (Roila et al. 2016). Previous MASCC/ESMO guidelines recommended the 5-HT3 receptor antagonist palonosetron plus dexamethasone for prophylaxis of acute nausea and vomiting; also, dexamethasone (p.o.) taken for several days has been recommended as preferred treatment for delayed emesis in MEC-treated patients (Roila et al. 2010). According to the MASCC/ESMO guidelines updated in 2016, a 5-HT3 receptor antagonist and dexamethasone is recommended for the prevention of acute emesis. Whether palonosetron in combination with dexamethasone is superior to other 5-HT3 receptor antagonists in MEC regimens is not clear, owing to a lack of comparative studies.

The effect of an NK-1 inhibitor arises from alleviation of the emetic effects of substance P on the central chemotrigger zone (Saito et al. 2009; Schmitt et al. 2014). Several large studies have shown that addition of aprepitant to a regimen containing granisetron or ondansetron and dexamethasone can significantly improve prevention of acute and delayed emesis for patients receiving highly emetogenic chemotherapy (HEC) (Stoltz et al. 2004; Curran and Robinson 2009).

According to the MASCC/ESMO guidelines, routine prophylaxis with an NK-1 receptor antagonist is not included for patients administered MEC. Conversely, the benefit of an aprepitant-containing triple antiemetic regimen for a broad range of MEC regimens has also been reported (Rapoport et al. 2010). The role of a NK-1 inhibitor with a second-generation 5-HT3 receptor antagonist as a prophylactic agent is also not clear.

Carboplatin in combination with third-generation antitumor agents is used widely as first-line chemotherapy for advanced non-small-cell lung cancer (NSCLC). Carboplatin is classified as a MEC agent, but carboplatin-containing combination chemotherapy, such as carboplatin plus pemetrexed, has high emetogenic potential (Ito et al. 2014). Antiemetic efficacy may not be satisfactory if only a 5-HT3 receptor antagonist and dexamethasone are administered for prevention of CINV in patients receiving carboplatin-based combination chemotherapy.

Considering the various mechanisms of actions of antiemetic drugs, appropriate combinations must be investigated. Chemotherapy agents categorized as MEC contain a wide spectrum of emetic potential, and there are few data on the emetic potential of combination chemotherapy. For prophylaxis of CINV, the effectiveness of combination treatment with an NK-1 inhibitor and first-generation 5-HT3 antagonist has been reported. However, the efficacy of a regimen comprising palonosetron, an NK-1 inhibitor, and dexamethasone for MEC has not been investigated thoroughly. We wished to assess the efficacy and feasibility of the strongest regimen (palonosetron, dexamethasone, aprepitant) as prophylactic treatment for patients receiving carboplatin-containing MEC.

## Patients and methods

This clinical trial was conducted as a phase-II, prospective, multicenter study of the East Japan Chesters Group. The study protocol was in accordance with the Declaration of Helsinki and registered at the University Hospital Medical Information Network Clinical Trials Registry as UMIN000017877. After approval from the Institutional Review Board of each participating institute, patients who had provided written informed consent were enrolled in seven hospitals between September 2011 and September 2014.

### Patient population

Chemotherapy-naïve patients, except those with lung cancer, receiving epidermal growth factor receptor tyrosine kinase inhibitors formed the study cohort. Eligible patients were adults (age > 20 years) with lung cancer confirmed by histology or cytology scheduled for a first course of a carboplatin-containing regimen (area under the curve for carboplatin = 5 or 6). Patients were also required to have adequate hematologic, hepatic and renal functions in laboratory tests, adequate oral intake and an Eastern Cooperative Oncology Group performance status (PS) of 0, 1, or 2.

Patients were excluded if they had any of the following: (i) malignancy of the central nervous system or other causes of nausea or vomiting unrelated to chemotherapy (e.g., gastrointestinal obstruction, massive ascites); (ii) active infection; (iii) uncontrolled pleural effusions; (iv) concomitant radiation therapy; (v) an emetic episode 24 h before initiation of chemotherapy; (vi) complications that prohibited dexamethasone use. Patients could not receive radiotherapy within 30 days before chemotherapy initiation.

Before the study commenced, an informed-consent form detailing the study procedure and its associated risks was explained to each patient. Any drugs with antiemetic efficacy other than the study drugs were not allowed before the study started, and were recorded on a medical chart if administered during the study period.

### Study design

Efficacy and safety of antiemetic therapy were assessed during an observation period from the administration of chemotherapy to day 7 (168 h). Patients were provided with a daily questionnaire to record any vomiting episode, rate their nausea, and to state dietary intake. Patients assessed their nausea and QOL using a 100-mm horizontal visual analog scale and with the Functional Living Index-Emesis (Martin et al. 2003). Tests of blood chemistry were undertaken before chemotherapy and days 6 or 7 to evaluate adverse effects. Adverse events, use of rescue therapy, and laboratory data were recorded on the medical chart. Patients were treated with triple antiemetic therapy comprising intravenous administration of 0.75 mg palonosetron and 9.9 mg dexamethasone on day 1, and oral administration of 125 mg aprepitant on day 1 followed by 80 mg on days 2 and 3. A dexamethasone dose ≤24 mg was allowed for patients being pre-medicated with paclitaxel according to the package insert.

### Objectives

Primary endpoint of the present study was the proportion of patients who did not experience vomiting and who did not require rescue medication [complete response (CR)] in the acute phase (0–24 h) and late phase (24–168 h). Secondary endpoint was the proportion of patients who experienced no vomiting episodes and no more than mild nausea without rescue medication [complete control (CC)]. Other endpoints were grade of nausea in acute and delayed phases, daily dietary intake, and QOL.

### Statistical analyses

The sample size was calculated to be ≥90 patients based on the assumption that the prevalence of a CR would be ≤70% (null hypothesis). The alternative hypothesis was that the prevalence of a CR would be >75% with an alpha value of 5% and power of 80%. Taking into account that some patients would be lost to follow-up, the total sample size required was determined to be ≥90 patients. Statistical analyses were done using SPSS (IBM, Armonk, NY, USA).

## Results

Ninety-two patients were enrolled between November 2010 and September 2014. As two patients were excluded from analyses because of a lack of efficacy data, 90 patients were included for analyses.

Patient characteristics are summarized in Table 1. Seventy-two patients were male and 18 patients were female (median age, 69 years). Eighty patients had NSCLC and 10 had small-cell lung cancer. Most of the patients had good PS (0 to 1), except for five patients with a PS of 2. Of the chemotherapeutic treatments that contained carboplatin, paclitaxel was the most common (given to 35 patients, 38.8%), whereas pemetrexed, TS-1, etoposide, and docetaxel were given to 24 (26.6%), 13 (15.5%), 12 (14.4%) and six (6.6%) patients, respectively.

**Table 1:** Patient characteristics

Sex	Male/female	72/18
Type of malignancy	Small-cell lung cancer	10
	Non-small-cell lung cancer	80
Performance status	0	35
	1	50
	2	5
Median age (range), years		69 (38–82)
Chemotherapy regimen		
	Carboplatin + paclitaxel	35
	Carboplatin + pemetrexed	24
	Carboplatin + TS-1	13
	Carboplatin + etoposide	12
	Carboplatin + docetaxel	6

### Efficacy

**Fig. 1:**
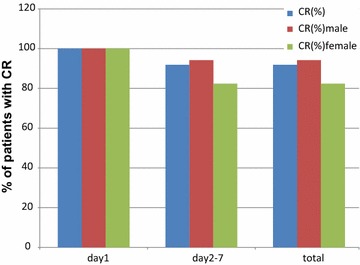
Complete response (CR) according to phase. This *bar graph* shows the percentage of patients achieving a CR 168 h after initiation of chemotherapy. A CR was defined as no vomiting and no requirement of rescue medication. *Blue bar* denotes all patients; *red bar* denotes male patients; *green bar* denotes female patients

**Fig. 2:**
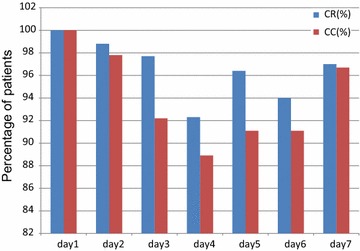
Time-course of a complete response (CR) over a 24-h period. This *bar graph* shows the percentage of patients achieving a CR and complete control (CC) on individual days after initiation of chemotherapy. *Blue* and *red bars* show the percentage achieving a CR and CC, respectively. A CR was defined as no vomiting and no use of rescue mediation. A CC was defied as no vomiting, no significant nausea, and no use of rescue medication

As no patients had a vomiting episode within 24 h from the start of chemotherapy, the prevalence of a CR in the acute phase was 100% (Fig. 1). Prevalence of a CR in the delayed phase (24–168 h) and overall (0–168 h) was 91.9%. Prevalence of a CR on individual days was 98.8% on day 2, 97.7% on day 3, 92.3% on day 4, 96.4% on day 5, 94.0% on day 6, and 97.0% on day 7 (Fig. 2).

**Fig. 3:**
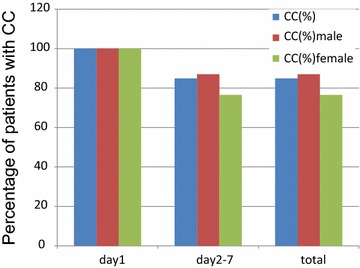
Complete control (CC) according to phase. This *bar graph* shows the percentage of patients achieving CC 168 h after initiation of chemotherapy. *Blue bar* denotes all patients; *red bar* denotes male patients; *green bar* denotes female patients

As no patients experienced vomiting or more than mild nausea, the prevalence of a CC in the acute phase was 100% (Fig. 3). Prevalence of a CC in the delayed phase and overall was 84.9%. Prevalence of CC on individual days was 97.8% on day 2, 92.2% on day 3, 88.9% on day 4, 91.1% on day 5, 91.1% on day 6, and 96.7% on day 7 (Fig. 2).

Male patients tended to have a higher prevalence of a CR and CC in the delayed phase compared with female patients. Prevalence of a CR in the delayed phase was 94.2% in male patients and 82.4% in female patients (Fig. 1). Prevalence of a CC in the delayed phase was 87.0% in male patients and 76.5% in female patients (Fig. 3).

**Fig. 4:**
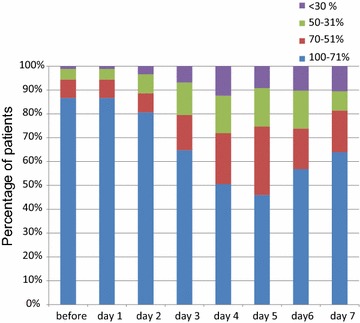
Meal intake over a 24-h period. This *bar graph* shows the percentage of patients according to food intake before and after initiation of chemotherapy. *Blue bar* denotes the percentage of patients who ate >71% of a meal served in hospital; *red bar*, 70–51%; *green bar*, 50–31%; *purple bar*, <30%

Meal intake on individual days is shown in Fig. 4. Before the start of chemotherapy, 86.6% of patients ate >71% of a meal served on day 1. There were significant decreases in dietary intake in the delayed phase. The proportions of patients who ate >71% of a meal served on days 4, 5 and 6 were 50, 44 and 55%, respectively.

### Safety

The most common adverse events exceeding grade 3 of the Common Terminology Criteria for Adverse Events (CTCAE) were neutropenia (5 cases), hypertension (5), diarrhea (1), and proteinuria (1), caused presumably by chemotherapy drugs (Table 2). No severe adverse events related to antiemetic treatment exceeding grade 3 of the CTCAE were observed, except for hypertension. The most common adverse event associated with antiemetic treatment was constipation (grade 1, 39 cases; grade 2, 4 cases). All patients were treated successfully by anti-constipation drugs and no specialized treatment (e.g., enema) was required to control constipation symptoms.

**Table 2:** Treatment-related adverse events

	Grade 1	Grade 2	Grade 3
Constipation	39	4	0
Hiccups	13	6	0
Hypertension	20	11	5
Cough	9	1	0
Insomnia	8	1	0
Fatigue	14	3	0
Increased LDH	20	1	0
Increased ALT	10	1	0
Increased AST	8	0	0
Increased BUN	19	0	0
Hypocalcemia	14	2	0
Hyperkalemia	6	1	0
Hyponatremia	17	0	0

Grade-4 treatment-related adverse events were not reported

*ALT* alanine aminotransferase, *AST* aspartate aminotransferase, *LDH* lactate dehydrogenase, *BUN* blood urea nitrogen

## Discussion

In Japan, some patients are hesitant to receive cancer chemotherapy because they are afraid of adverse effects such as CINV. Much progress has been made in supportive care, but clinical experts frequently underestimate the severity of nausea and vomiting, and many patients suffer from CINV without optimal management of symptoms (Di Maio et al. 2015). The aim of antiemetic therapy should be to minimize or eliminate CINV in all cancer patients. However, recommended treatments for CINV vary among guidelines, and half of patients receiving MEC are afflicted by CINV (Ihbe-Heffinger et al. 2004). An investigational study to establish optimal antiemetic treatment for MEC is thus warranted.

Several studies have reported that combination treatment using a 5-HT3 receptor antagonist, an NK-1 receptor inhibitor, and dexamethasone are useful for prevention of the CINV caused by HEC (Miura et al. 2013; Longo et al. 2011). Triple therapy comprising palonosetron, aprepitant and dexamethasone seems to be the strongest antiemetic treatment. Miura et al. (2013) reported on the efficacy of triple treatment for CINV in lung-cancer patients receiving HEC. Prevalence of a CR and CC overall was 81.1 and 66.7%, respectively, and treatment carried a good safety profile. None of the severe adverse events exceeded grade 3 of the CTCAE. Mild constipation that was readily manageable was reported to be the most common adverse effect. Considering the excellent profile of that treatment, triple therapy should be investigated as prophylaxis against the CINV observed with MEC.

Warr et al. (2005) demonstrated that addition of aprepitant to ondansetron and dexamethasone enhances the antiemetic effect of MEC using cyclophosphamide plus doxorubicin or epirubicin (AC). However, the prevalence of a CR was only 51% in the aprepitant group and only 42% in the control group over 5 days. Grunberg et al. (2009) reported on the efficacy of a triple regimen comprising palonosetron, dexamethasone and aprepitant for prevention of acute and delayed CINV caused by MEC. Prevalence of a CR was relatively good in the acute phase (76%), but was not satisfactory overall (51%). Those results are confusing because most patients were treated with an AC regimen, which is now classified as HEC in the MASCC/ESMO guidelines. The value of antiemetic treatment should be evaluated more strictly according to the respective characteristics of each chemotherapy drug.

The MASCC/ESMO guidelines updated in 2016 state that addition of an NK-1 inhibitor to a 5-HT3 receptor antagonist and dexamethasone is recommended to prevent carboplatin-induced acute nausea and vomiting (Roila et al. 2016). Ito et al. (2014) reported a randomized phase-2 trial that compared standard antiemetic therapy with a 5-HT3 receptor antagonist and dexamethasone with aprepitant add-on triple antiemetic therapy in patients with NSCLC who received carboplatin-based first-line chemotherapy. The aprepitant group showed a better overall CR of 80.3% compared with that of 67.2% for the control group. Tanioka et al. (2013) reported on a randomized study of aprepitant in women receiving MEC comprising mainly a carboplatin- or irinotecan-containing regimen. Prevalence of a CR overall was superior, but not significantly higher, in the aprepitant, granisetron and dexamethasone group than in the placebo, granisetron and dexamethasone group (aprepitant group, 62.2%; placebo group, 52.1%). The authors concluded that the addition of aprepitant seemed to be effective and that an antiemetic regimen equivalent to that used for HEC was well tolerated and seemed to be more effective for CINV prevention in women receiving MEC. Use of palonosetron instead of granisetron might improve delayed CINV because American Society of Clinical Oncology guidelines recommend palonosetron as the preferred 5-HT3 receptor antagonist for a non-AC MEC regimen (Basch et al. 2011). However, no clinically relevant differences between palonosetron and other 5-HT3 receptor antagonists have been demonstrated by randomized trials for a non-AC MEC regimen. There is a lack of evidence of comparative studies in MEC agents demonstrating an advantage of the use of palonosetron with respect to other 5-HT3 receptor antagonists. According to the general rule of MASCC/ESMO guidelines, a benefit of ≥10% is sufficiently clinically meaningful to warrant a change in guidelines. In our study, triple antiemetic therapy comprising palonosetron, dexamethasone, and aprepitant achieved a CR of 100 and 91.9% in acute and delayed phases, respectively. These data suggest that triple antiemetic therapy could increase the prevalence of a CR by ≥10%. Hence, the efficacy of triple therapy should be investigated.

Results of antiemetic studies in patients treated with MEC are summarized in Table 3. Celio et al. (2011) reported that the prevalence of a CR in the acute phase and delayed phase was 88.6 and 68.7%, respectively, in patients treated with palonosetron and dexamethasone as antiemetic treatment. Prevalence of a CR in other reports treated MEC not specified regimen tended to be equal or less than in that report. In the present study, the chemotherapy regimens administered to enrolled patients were based on carboplatin. Prevalence of a CR in the acute phase and delayed phase was excellent (100 and 91.9%, respectively). Prevalence of a CR was remarkably better than that in other studies stating that palonosetron, dexamethasone and aprepitant is the strongest antiemetic treatment that produces excellent antiemetic effects against MEC using a carboplatin-based regimen.

**Table 3:** Summary of studies focusing on the efficacy of antiemetic therapy in patients receiving MEC

Author	Regimen (mg)	Acute CR (%)	Delayed CR (%)	Overall CR (%)	Chemotherapy
Eisenberg et al. (2003)	Palo (0.25)	63	54	46	MEC
	Palo (0.75)	57.1	56.6	47.1	
Celio et al. (2011)	Palo (0.25) + Dex (8) day 1	88.6	68.7	67.5	MEC
	Palo (0.25) + Dex (8) days 1-3	84.3	77.7	71.1	
Tanioka et al. (2013)	Gra (1) + Dex (12) + Ap	97.8	62.2	62.2	CBDCA-based
	Gra (1) + Dex(12)	95.7	52.2	52.2	
Hesketh et al. (2016)	Gra (2) day1-3 + Dex (20) + Rol (180)	91.7	82.3	80.2	CBDCA-based
	Gra (2 mg) days1-3 + Dex (20 mg)	88.0	65.6	64.6	
Yahata et al. (2016)	Gra (1/4) + Dex (20) + Ap	94.0	63.6	61.6	CBDCA + PAC
	Gra (1/4) + Dex (20)	90.4	49.3	47.3	
Present study	Palo (0.75) + Dex (9.9) + Ap	100	91.9	91.9	CBDCA-based

Doses of Ap were the standard doses recommended by various guidelines such as 125 mg on day 1 and 80 mg on days 2 and 3. “MEC” in the chemotherapy column indicates that the MEC was not specified

*CR* complete response, *Palo* palonosetron, *Gra* granisetron, *Dex* dexamethasone, *Ap* aprepitant, *Rol* rolapitant, *MEC* moderately emetogenic chemotherapy, *CBDCA* carboplatin, *PAC* paclitaxel

Hesketh et al. (2016) reported that addition of rolapitant to a 5-HT3 receptor antagonist and dexamethasone provided patients who received carboplatin-based chemotherapy with superior protection against CINV. Prevalence of a CR was significantly higher with rolapitant treatment than that achieved with the control overall and in the delayed phase. Prevalence of a CR in the acute phase was 91.7%, which is close to that obtained in the present study.

The mechanism of action of delayed CINV is not entirely understood, but it is considered to be different to that of acute CINV. Delayed CINV is thought to arise through the effects of substance P in the central chemotrigger zone (Curran and Robinson 2009). Even though the introduction of a first-generation 5-HT3 receptor antagonist has elicited significant improvements in prophylactic care against CINV, control of delayed emesis remains an unaddressed need. Schmoll et al. (2006) reported that a 5-HT3 receptor antagonist administered on multiple occasions may be more effective than a 5-HT3 receptor antagonist administered once for control of delayed CINV. Palonosetron is a second-generation selective antagonist against the 5-HT3 receptor that has an ≈100-fold stronger binding affinity for the 5-HT3 receptor compared with first-generation agents, and an extended plasma elimination half-life of ≈40 h (Aporo et al. 2006). If the long half-life of palonosetron compensates for the effect of the delayed phase, a single dose of palonosetron could be more convenient than multiple doses of a first-generation 5-HT3 receptor antagonist. Roscoe et al. (2012) presented data from a double-blind randomized clinical trial for control of delayed nausea. They reported that addition of dexamethasone on days 2 and 3 reduced CINV in the delayed phase. Conversely, Celio et al. (2013) demonstrated that a dexamethasone-sparing regimen is not associated with a significant loss in overall antiemetic protection in women undergoing AC if palonosetron is used as an antiemetic.

The beneficial effect of an NK-1 inhibitor for control of delayed CINV is also controversial. Efficacy of addition of aprepitant for control of delayed CINV has been reported to be identical to that elicited by addition of prochlorperazine (an antipsychotic drug that acts on dopaminergic receptors at the chemoreceptor trigger zone) (Roscoe et al. 2012). Olanzapine is another antipsychotic drug whose effect is manifested through blockade of multiple neurotransmitter receptors. Navari et al. (2016) reported on a randomized, double-blind, phase-3 trial comparing olanzapine with placebo, in combination with dexamethasone, aprepitant or fosaprepitant, and 5-HT3 receptor antagonist in patients receiving cisplatin or AC. Olanzapine significantly improved nausea prevention, as well as the prevalence of a CR, among patients receiving HEC. They concluded that olanzapine was a very promising candidate for control of acute and delayed CINV in patients receiving HEC.

In the present study, the prevalence of a CR and CC in the delayed phase was excellent (91.9 and 84.9%, respectively), but dietary intake decreased significantly on days 3 to 6. Admittedly, there is room for improvement in treatment of delayed nausea, but the triple regimen has a tremendous effect on patients receiving MEC based on carboplatin.

This clinical trial assessed the efficacy of triple treatment only in the first cycle of chemotherapy. However, sustained efficacy of the same antiemetic regimen has been reported throughout repeat chemotherapy cycles in a population receiving HEC (Sakai et al. 2008). Control of CINV in the first cycle is important because the CINV of subsequent cycles and anticipatory emesis are related to the degree of CINV of the first cycle (Chan et al. 2015). Maximally effective antiemetics as first-line therapy should be used rather than withholding more effective drugs for subsequent use at the time of antiemetic failure.

Being female and young is considered to contribute to the variance in acute nausea and severity of delayed nausea when chemotherapy is administered (Molassiotis et al. 2014). In our study, the prevalence of a CR and CC was consistently better for male patients, but the number of patients was not sufficient to power statistical analyses. Future clinical trials should consider differences in CINV prevalence according to sex. The cost of medication is another important issue: the antiemetic drugs evaluated in our study are indicated and covered by insurance from the Japanese government.

## Conclusions

We demonstrated the excellent effect of a triple regimen comprising palonosetron, dexamethasone and aprepitant as prophylactic care for patients treated with a carboplatin-containing regimen. This triple regimen did not enhance adverse effects. If the “ultimate” goal in CINV research is absence of CINV in patients undergoing chemotherapy, use of the triple regimen described here could be a candidate.

## Abbreviations

CINV: chemotherapy-induced nausea and vomiting
CR: complete response
CC: complete control
5-HT3: 5-hydroxytryptamine-3
NK-1: neurokinin-1
QOL: quality of life
AC: cyclophosphamide plus doxorubicin or epirubicin
NSCLC: non-small-cell lung cancer
MEC: moderately emetogenic chemotherapy
HEC: highly emetogenic chemotherapy
